# The Influence of Nanomaterials on the Thermal Resistance of Cement-Based Composites—A Review

**DOI:** 10.3390/nano8070465

**Published:** 2018-06-26

**Authors:** Pawel Sikora, Mohamed Abd Elrahman, Dietmar Stephan

**Affiliations:** 1 Building Materials and Construction Chemistry, Technische Universität Berlin, Berlin, Gustav-Meyer-Allee 25, 13355 Berlin, Germany; abdelrahman@tu-berlin.de (M.A.E.); stephan@tu-berlin.de (D.S.); 2 Faculty of Civil Engineering and Architecture, West Pomeranian University of Technology, Szczecin, Al. Piastow 50, 70-311 Szczecin, Poland; 3 Structural Engineering Department, Mansoura University, Elgomhouria St., Mansoura 35516, Egypt

**Keywords:** nanomaterials, review, cement-based composites, concrete, elevated temperature, fire resistance, mechanical properties, microstructure

## Abstract

Exposure to elevated temperatures has detrimental effects on the properties of cementitious composites, leading to irreversible changes, up to total failure. Various methods have been used to suppress the deterioration of concrete under elevated temperature conditions. Recently, nanomaterials have been introduced as admixtures, which decrease the thermal degradation of cement-based composites after exposure to high temperatures. This paper presents a comprehensive review of recent developments related to the effects of nanoparticles on the thermal resistance of cementitious composites. The review provides an updated report on the effects of temperature on the properties of cement-based composites, as well as a detailed analysis of the available literature regarding the inclusion of nanomaterials and their effects on the thermal degradation of cementitious composites. The data from the studies reviewed indicate that the inclusion of nanoparticles in composites protects from strength loss, as well as contributing to a decrease in disruptive cracking, after thermal exposure. From all the nanomaterials presented, nanosilica has been studied the most extensively. However, there are other nanomaterials, such as carbon nanotubes, graphene oxide, nanoclays, nanoalumina or nano-iron oxides, that can be used to produce heat-resistant cementitious composites. Based on the data available, it can be concluded that the effects of nanomaterials have not been fully explored and that further investigations are required, so as to successfully utilize them in the production of heat-resistant cementitious composites.

## 1. Introduction

Concrete is a well-understood material and for most application purposes has a satisfying endurance to elevated temperatures. It has been used successfully in various engineering structures, providing satisfactory dimensional stability to structures in cases of fire [1], although its properties deteriorate when exposed to elevated temperatures. Alteration of the microstructure of cement-based composites occurs from the very beginning of the heating process, with cement paste already exhibiting some damage even after heating up to 105 °C (the standard temperature for the drying of materials) [2]. However, temperatures up to 200–300 °C can still be considered relatively harmless to the stability of cementitious composites [3]. Nonetheless, exceeding certain temperatures, as well as certain exposure times, can lead to irreversible changes in concrete, even leading to structural collapse. Resistance of concrete to high temperatures is highly related to the thermal conductivity and specific heat that are influenced by many factors including the aggregate type, mixture composition, and moisture content [1]. Concrete provides lasting protection to reinforcing steel (which is sensitive to high temperature), however, due to its thermal conductivity non-linear temperature distribution in the concrete element occurs [4]. As a result, thermal transients propagate progressively within the concrete element, leading to significant thermal gradients and thus, causing undesirable damage to the concrete [4].

Concrete is widely used as a material for high-rise buildings, tunnels, drilling platforms, as well as nuclear facilities (having both satisfactory thermal and shielding properties) [5,6,7,8]. However, recent accidents involving existing structures have revealed that there is still a strong need to continue studies, in order to understand the effects of high temperature on cement-based materials, as well as to find methods for improving their thermal resistance. However, understanding the performance of concrete under elevated temperature conditions is a complex issue, due to its heterogeneous nature (consisting of cement paste and aggregates); thus, response to stress is not only dependent on the response of individual components, but also on the interaction between those components. For this reason, deterioration in the mechanical and physical properties of concrete depends on individual physicochemical changes in the cement paste and aggregates, as well as on (thermal) incompatibility between the aggregate and surrounding cement paste [9]. Therefore, to provide satisfactory thermal resistance for cement-based composites working under or exposed to elevated temperature conditions, mix design, including proper proportions and constituents, is a key factor when designing the material [9]. Nevertheless, the compatibility of the constituents should be provided not only when unheated materials are tested, but also within a range of heating temperatures.

The choice of proper aggregate plays a crucial role in providing a satisfactory thermal resistance to mortars and concretes, as aggregate makes up 60 to 80 vol. % of the concrete [2,9]. As such, aggregates with low mass loss and a low thermal strain coefficient of expansion, along with negligible residual strains, are clearly preferred in the production of fire-resistant concretes [2,10]. Moreover, the rough angular surfaces of aggregates (which improve the physical cement paste-aggregate bond), as well as the presence of reactive silica (which improves chemical bonds), are desirable features of aggregates [9]. In general, aggregates usually remain stable up to 500–600 °C, although some siliceous aggregates, such as flint [9,11], are stable only up to 300–350 °C [2,12]. It has been reported that siliceous aggregates (i.e., quartzite, granite), have worse thermal resistance than carbonatic aggregates (such as limestone or dolomite) [3,5,11]. This is attributable to the higher thermal expansion of carbonatic aggregates, as well as to the quartz crystal transition, which takes place at around 573 °C (low to high quartz), resulting in volume expansion [11]. In the case of calcareous aggregates, a temperature range of 700 °C to 900 °C seems to be disruptive, as a result of the decomposition of calcium carbonate; CaCO_3_ decomposes into CaO (lime) and CO_2_ (carbon dioxide) [2,5,13].

In distinction to the expansion process of aggregates, when heated, cement paste generally exhibits shrinkage, due to the chemical and physical loss of water, when exposed to elevated temperatures, although up to 200 °C, a slight expansion might occur. Hydrated cement paste is mainly composed of calcium-silicate-hydrate (C–S–H) gel (the primary binding phase), calcium hydroxide (CH) and calcium sulfoaluminates (ettringite and monosulphate), which consist of 50–60 vol. %, 20–25 vol. % and 15–20 vol. % of solids, respectively [5].

The thermal resistance of cement paste is governed by many factors, in which the most important are the water to cement (w/c) ratio, the C/S (calcium oxide/silicon dioxide ratio), as well as the quantity of portlandite (CH). A paste containing a low C/S ratio and thus a low CH content is more desirable for obtaining heat-resistant cement paste [3,9,13].

**Figure 1:**
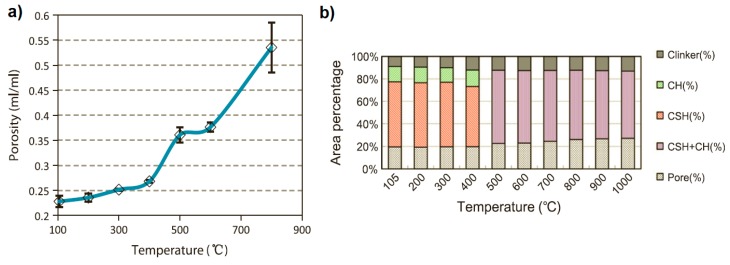
The effect of temperature on the change of total porosity (**a**) and the area percentage of each phase (**b**) of heated Portland cement paste after cooling. Reproduced with permission from [15]. Copyright Elsevier, 2013.

In general, the degradation process of cement paste occurs from the very beginning of the heating process, although up to 300 °C the changes are considered to be relatively small and reversible, being thus recoverable by the so-called “rehydration process”. At 80 °C, the decomposition of ettringite begins, as well as the evaporation of physically bound water. Firstly, the capillary and free water not influenced by the Van der Walls attraction forces, evaporate; afterward, the loss of physically and chemically combined water, associated with the C–S–H phase, occurs [3,13]. The C–S–H decomposition process starts at the very beginning of the heating process, resulting in a reduction of its volume, which turns to an increment in total porosity as well as a coarsening of the pore structure [2,13]. However, up to 300 °C, no dramatic changes in porosity are observed, but when the temperature exceeds 300 °C, the porosity increases significantly [13]. Figure 1 presents the change in total porosity (up to 800 °C) and the area percentage of each phase of heated Portland cement paste when heated up to 1000 °C. A similar relation between temperature increment and the coarsening of the pore structure can be observed in the case of concrete. Using the mercury intrusion porosimetry method (MIP), Haridharan et al. [14] have analyzed the porosity of concretes, which were exposed to elevated temperature and then cooled quickly or slowly. With a slow cooling process, porosity increased from an initial 19.24 vol. % to 22.34 vol. %, 25.32 vol. %, and 32.54 vol. % after exposure to 200, 400 and 600 °C, respectively. Likewise, after quick cooling, the porosity increased to 22.17 vol. %, 32.87 vol. %, and 44.54 vol. % after exposure to 200, 400 and 600 °C, respectively.

In addition to the alternation of the pore structure while heating, when the temperature exceeds 200 °C the first cracks in the cement matrix can be noticed; however, a significant increment in cracks is observable only when the temperature exceeds 400 °C. Above a temperature of 400 °C, the number of cracks increases drastically with temperature [3,5]. Moreover, when the temperature rises, thermal incompatibilities between aggregate and cement paste arise, leading to propagation of micro-cracking in the interfacial transition zone (ITZ) between the cement paste and the aggregate [3], resulting in significant strength loss. 

**Figure 2:**
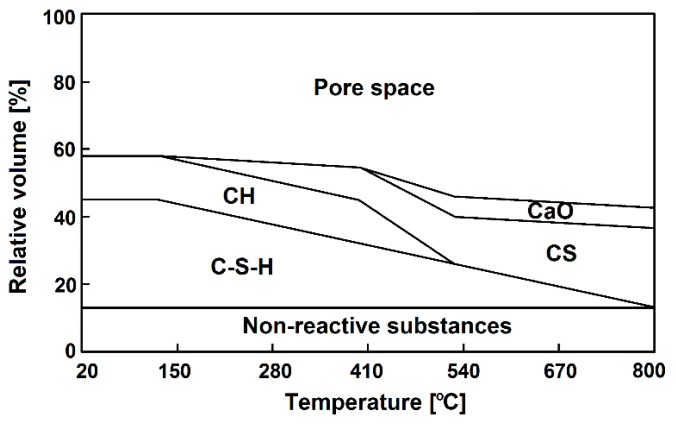
The effect of temperature on the change of phase composition of cement paste with w/c = 0.67. Reproduced with permission from [17]. Copyright Elsevier, 2009.

A turning point in the properties of cement-based composites occurs in the range of 400 °C to 550 °C. At these temperatures, decomposition of CH occurs [2,3,13]. This process is not itself critical regarding the strength loss of concretes; however, as a result of a rapid lime rehydration process (when samples are water cooled or exposed to humidity), portlandite is formed again, resulting in expansion, which turns to severe cracking and strength loss in samples [3]. Therefore, cement blends produced with lower C/S ratios possess lower CH contents, thus decreasing the severe cracking phenomenon [9]. As reported by Arioz [16], exposure to a temperature of 500 °C causes irreversible changes in concretes. A further temperature increment leads to the second phase of C–S–H gel decomposition at 560 °C and formation of β-C_2_S at 600–800 °C, as well as the decomposition of poorly crystalized CaCO_3_ to CaO and CO_2_ [2,3,5,13]. A temperature of 600 °C seems to be critical for concrete, resulting in an extreme increase in cracks observed on the surface of the concrete, as well as significant growth of porosity, resulting in a dramatic strength loss in the concrete [5,9]. After exposure to 600 °C, concrete loses its load-bearing capacity, and can thus be considered structurally useless. A graphical representation of the change of phase composition in hydrated cement paste (w/c = 0.67), as a function of heating temperature, is presented in Figure 2. Table 1 summarizes the effects of temperature on the properties of concrete.

Various methods for improving the thermal resistance of cement-based composites exist: the use of proper aggregates [11], the use of various fibers (especially polypropylene fibers—PP) [18,19], the application of surface coatings [20], and the incorporation of supplementary cementitious materials (SCMs) [3,5,21]. It has been reported that the inclusion of SCMs, such as fly ash (FA) or ground granulated blast-furnace slag (GGFBS), can contribute to strength improvements at lower temperatures (up to 400 °C), as well as to minimizing strength loss up to 600 °C, as compared to plain Portland cement concrete. In addition, due to the pozzolanic activity of SCMs resulting in an extra amount of C–S–H produced, along with a reduced amount of CH, a reduction in the surface cracks of concretes is observed. Thus, the total porosity and average pore volume become lower than that of plain cement concrete [3,5]. Furthermore, the abovementioned SCMs contribute to a decrease in the risk of explosive spalling. Silica fume (SF) is a type of SCM which leads to an increase in the risk of spalling. This effect is attributable to the filler effect and the pozzolanic activity of SF, resulting in the production of a cement matrix with low permeability. Hence, as a result of high vapor pressure during concrete heating, the risk of thermally-induced explosive spalling increases [3,5,21,22].

In the last decade, the use of nanomaterials, as admixtures for improving the thermal resistance of cementitious composites, has gathered the noticeable attention of researchers. The use of nanomaterials has been widely promoted due to their superior reactivity, as compared to similar materials available at the micro-scale [23,24,25]. Nanomaterials affect the properties of cementitious materials in two ways: by their hydration seeding effect and their filling effect [23,26,27]. Due to their small size, nanomaterials act as seeds during the precipitation process, accelerating the hydration process and enabling C–S–H gel formation around the nanomaterials. In addition to their chemical effect, nanomaterials exhibit a particular physical effect: the so-called nano-filling effect, which leads to densification of the pore system [23]. As a result, the pore structure becomes more compact and homogenous, which translates to a significant improvement in mechanical properties and durability. However, the incorporation of nanomaterials might lead to noticeable consistency decrement [24] and undesirable compaction (as a result of high surface area to volume ratio of nanoparticles) of cement matrix resulting in cement matrix of low permeability, thus increasing susceptibility to cracking during thermal load (as in case of SF-incorporated concrete) [28].

Even though there are numerous review papers related to the effects of nanoparticles on cement-based composites in the ambient (unheated) state [23,24,29,30,31,32,33], there is a lack of review papers summarizing the effects of nanomaterials on the performance of cementitious composites under elevated temperature conditions. As such, this paper aims to summarize recent developments in this field, as well as to address the possible directions which are necessary for developing heat-resistant nanomodified cementitious composites.

## 2. The Effect of Nanosilica on the Thermal Resistance of Cement-Based Composites

Among all the nanomaterials used to modify the properties of cement-based composites, nanosilica (NS) has most attracted researchers’ attention. This interest can be attributed to NS’s superior reactivity, as compared to other SCMs. NS fills the spaces between the particles of C–S–H gel, acting as a nano-filler and refining its microstructure. Also, due to its high pozzolanic activity, nanosilica reacts with CH, producing more C–S–H, which results in a denser cement matrix and thus improved strength and durability of cement-based composites [29]. The effect of nanosilica on the mechanical properties and durability of cementitious composites has been comprehensively revised by many authors [23,24,29,30,31,33,34].

Studies undertaken by various researchers have shown that nanosilica can be utilized in cement-based composites working at elevated temperatures. The available studies related to the effects of NS on the thermal resistance of cement-based composites are summarized in Table 2. The inclusion of nanosilica in the mix improves the resistance of cementitious composites to elevated temperatures, since such composites retain their high residual compressive strength and demonstrate reduced crack length and width, as compared to plain samples. It has been reported that nanosilica improves the chemical stability of C–S–H (due to a reduction of calcium leaching from C–S–H) and increases the average silicate chain length of C–S–H and the volume of high-density C–S–H. As a result, the thermal degradation of the C–S–H phase during heating can be hindered [35,36,37]. Moreover, in the lower temperature range (up to approximately 300–400 °C), due to the high pozzolanic activity of NS, nanoparticles promote the hydration of anhydrous cement grains during the internal autoclaving process and as a result more C–S–H is produced. Subsequently, a higher strength increment is obtained for samples containing NS than for plain cementitious composites. Also, due to the consumption of portlandite and the formation of additional C–S–H, nanosilica contributes to the filling of some open pores, and therefore a reduction of surface cracks can be observed after thermal exposure [28,38]. Furthermore, Kumar et al. [39] have reported that the incorporation of NS decreases the thermal conductivity of concretes and that heat transfer in NS-modified specimens is thus delayed. It means that more time is required to reach the desired temperature at the core of specimens so that the rate of thermal degradation of specimens is delayed, as compared to plain specimens.

### 2.1. The Effect of Nanosilica on Mass Loss

Heikal et al. [40] have analyzed the mass loss (up to 950 °C) of ordinary Portland cement (OPC) pastes containing 1 and 4 wt. % of NS and cement pastes containing 25, 50 and 65 wt. % of cement replaced with blast-furnace slag (GGBS) and a fixed amount of NS—4 wt. %. It was found that in the case of OPC pastes, mass loss was higher for samples without an NS admixture, as a result of the higher CH amount in the cement paste. Incorporation of 1 wt. % or 4 wt. % of NS contributed to a decrement in the amount of calcium hydroxide (CH) and calcium carbonate (CC^−^) in cement pastes, but the lowest mass loss of OPC cement pastes was reported for the sample containing 1 wt. % of NS. When GGBS and 4 wt. % of NS was introduced to the cement paste, a higher mass loss of GGBS-incorporated specimens was reported, in the range of 200–400 °C, as compared to OPC pastes. This was attributed to the activation of GGBS (as a result of self-autoclaving conditions), nano-filling, and the high pozzolanic activity of NS. This phenomenon resulted in the production of an additional C–S–H phase from the liberated CH. However, when the temperature exceeded 400 °C, the mass loss of GGBS-incorporated cement pastes was generally lower than that of OPC pastes. Maheswaran et al. [43] have analyzed the mass loss (based on thermogravimetric analysis) of cement pastes containing 20 wt. % of cement replaced with lime sludge and an addition of nanosilica (1.5 wt. % and 3 wt. %) with a fixed w/b = 0.4. After exposure to temperatures up to 400 °C, the mass loss of samples containing NS was slightly higher than that of plain samples, while in the case of exposure to a temperature range of 400 °C to 600 °C, the mass loss of samples containing NS was lower, as a result of CH consumption by the NS. 

In another study [28], the effects of NS admixture in amounts from 1–5 wt. %, on the mass loss of cement mortars (w/c = 0.5) containing three different types of aggregates (quartz, magnetite and barite), were evaluated. The samples were subjected to elevated temperatures of up to 800 °C. No significant effect of the NS content or heating temperature on the mass loss of cement mortars was observed, with the mass loss being highly related to the type of aggregate used. Bastami et al. [46] have evaluated the effects of temperature (up to 800 °C) on the mass loss of two types of high-strength concretes (HSCs), with w/b = 0.25, containing fixed amounts (30 kg/m^3^ or 60 kg/m^3^) of SF or SF/NS. In NS-modified concretes, SF was replaced with NS in amounts of 1.5, 3, and 4.5% by mass of cement (1.41, 2.83 and 4.2 wt. % of binder mass, respectively). The results of these studies were inconclusive, although at up to 400 °C the mass loss of was slightly lower or negligible for samples containing NS. After exposure to 600 °C and 800 °C, in comparison with plain concrete, a slightly lower mass loss was observed for concretes containing 30 kg/m^3^ of SF/NS mixture, while in concrete specimens containing 60 kg/m^3^ of SF/NS mixture, the mass loss increased. 

In a study presented by Shah et al. [47], the mass loss of three HSCs (w/b ranging from 0.25 to 0.3), containing 5 wt. % of NS, 10 wt. % of SF or a mixture of 5 wt. % NS and 5 wt. % SF, exposed to 200, 500 and 800 °C, was evaluated. After exposure to 200 °C, the mass loss values for all concretes tested was relatively similar, varying from 2.47 to 3.17%. After exposure to 500 °C, samples containing NS exhibited a higher mass loss than SF-modified specimens, although the highest mass loss was reported for samples containing a mixture of SF and NS. After exposure to 800 °C, the lowest mass loss was reported for NS-modified samples, while mass loss dramatically increased for samples containing SF and a SF/NS mixture. Rathi and Modhera [48] have analysed the thermal resistance of HSCs with cement replacements; from 10 up to 30 wt. % of FA (in increments of 5 wt. %) and with 1 to 5 wt. % of NS (in 1 wt. % increments). For comparison, plain concretes without NS or FA were prepared. The authors reported that in all the mixtures tested and under all testing temperatures (from 100 to 800 °C), the mass loss was found to be smallest for concretes containing 3 wt. % of NS. Kumar et al. [39] have evaluated the properties of two HSCs containing 0 and 3 wt. % NS (w/c = 0.29), exposed to 200, 400, 600 and 800 °C. The authors reported that >200 °C, the mass loss of NS modified concrete was decreased slightly, as a result of the lesser amount of free water available to evaporate from the concrete (consumed as a result of the formation of a higher quantity of hydration products). As a result, after exposure to 400 °C, the mass loss of NS-modified concretes increased slightly, because of the dehydration of the higher amount of C–S–H, formed as a result of the presence of NS. Further exposure to 600 and 800 °C, resulted in a slightly lower mass loss of NS-modified concretes, as a result of delayed thermal degradation, attributed to the decreased thermal conductivity of NS-modified concretes.

From the references cited above, it can be concluded that the effect of NS on the mass loss of cement-based composites is still controversial and is highly dependent on mix compositions as well as on the type of composite tested (e.g., paste, mortar or concrete). It is difficult to draw a clear conclusion about the influence of the incorporation of nanosilica on mass loss or the stability of cement composites when subjected to high temperatures. Consequently, this issue requires further investigation, to determine a distinct trend regarding the performance of various dosages of NS on the stability of different cement-based materials.

### 2.2. The Effect of Nanosilica on the Mechanical Properties of Cement-Based Composites

#### 2.2.1. Flexural Strength

The effect of NS on the flexural strength of cement-based composites is still in dispute, as flexural strength is highly sensitive to micro-cracking during heating [28]. In work undertaken by Ibrahim et al. [45], the effect of NS (in the amounts of 0, 2.5, 5 and 7.5 wt. %) on the flexural strength of cement mortars, containing OPC and PP fibers exposed to high temperatures, was determined. Samples were cured for 3, 7, and 28 days and then exposed to 400 and 700 °C. In general, the authors reported an improvement in flexural strength (after all curing periods) in unheated cement mortars, when NS was present. After exposure to 400 °C, the flexural strength of all the cement mortars decreased, as a result of thermally induced stress leading to increased cracking and pressure in gel pores. However, samples containing 2.5 wt. % and 5 wt. % of NS (after 28 days of curing) exhibited higher flexural strength retention than plain control samples. On the other hand, in the case of samples containing 7.5 wt. % of NS, flexural strength was slightly lower than that of the control samples. After exposure to 700 °C, all the samples lost almost all of their strength, and no significant effect of NS was observed. In another study, carried out by the same authors [44], the effect of replacing FA with NS (in the amounts of 0, 2.5, 5, and 7.5 wt. %), on the properties of high-volume fly ash cement mortars (25, 35 and 45 wt. % of FA) exposed to high temperature, was evaluated. Samples were exposed to 400 and 700 °C. Similarly to the abovementioned studies [45], positive effects of NS-modified samples on mechanical properties (after 3,7 and 28 days of curing), in unheated states, were observed. The study showed that the partial replacement of FA with NS contributes significantly to the retention of flexural strength loss, after exposure to 400 °C. The best performance was exhibited by cement mortar containing a mixture of 37.5 wt. % FA and 7.5 wt. % NS (total 45 wt. %). After exposure to 700 °C, these samples lost most of their flexural strength, with their strength values varying between 20–30% of the initial strength. 

In distinction to the abovementioned works, other studies have reported rather negligible effects of NS on flexural strength [28,49,51]. Yan et al. [51] have analyzed the effect of NS on the flexural strength of steel-fiber reinforced concrete (SFRC), exposed to temperatures of up to 800 °C. They reported an increment in the initial flexural strength of concrete containing NS, as well as a slightly higher flexural strength after exposure to 200 °C, compared to plain SFRC. Beyond 200 °C, the effect of NS seemed to be negligible. In another study [49], the effect of 200, 400, and 600 °C on the flexural strength of two types of concretes with w/c = 0.25 (containing 350 and 450 kg/m^3^ of cement), with the addition of 15 wt. % of SF and 1–5 wt. % of NS, was examined. Adding NS improved the flexural strength of unheated concrete specimens, although no significant changes (<10%) in residual flexural strength, between specimens exposed to temperature, were reported. A negligible effect of NS addition (1–5 wt. %), on the flexural strength of cement mortars containing different types of aggregates (quartz, magnetite, and barite) exposed to temperatures up to 800 °C, has also been reported in a study undertaken by Horszczaruk et al. [28]. A slight variation (decrement) in the flexural strength of cement mortars containing barite aggregate and nanosilica was reported, although this effect was attributed to the properties of the barite aggregate, which has low thermal resistance and cracking potential under thermal stress.

It is difficult to draw any clear conclusions, regarding the effects of NS on the flexural strength of cementitious composites, from the works cited above. While some authors have reported flexural strength improvements [44,45] in the presence of NS under elevated temperatures, others have reported only negligible effects [28,49,51]. As such, further work is required.

#### 2.2.2. Tensile Strength

**Figure 3:**
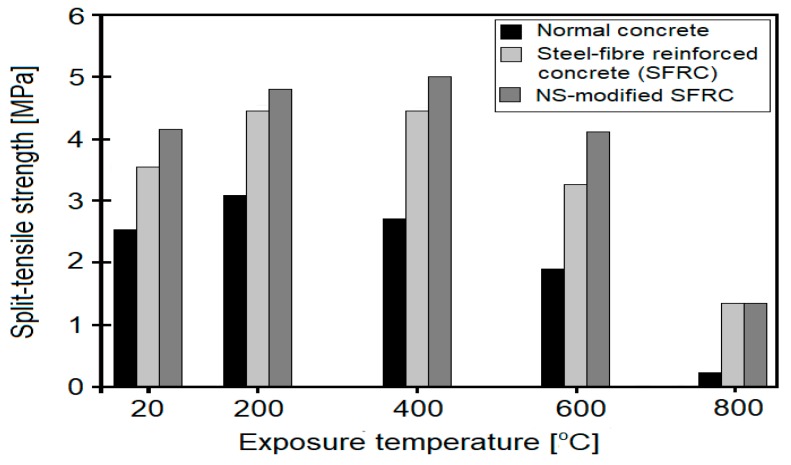
Split-tensile strength of normal concrete, steel-fiber reinforced concrete (SFRC) and SFRC modified with NS, as a function of temperature. Redrawn from [51].

Tensile and split-tensile strength have been analyzed in several works [39,46,50,51]. In the work done by Bastami et al. [46], the effect of temperature (up to 800 °C) on the tensile strength of HSC (w/b = 0.25), containing 30 kg/m^3^ of SF replaced with NS in the amounts of 0, 1.5, 3 and 4.5 wt. % (by mass of cement), was determined. The best performance was observed for samples containing 4.5 wt. % of nanosilica. NS-modified samples exhibited higher tensile strength retention, as compared to plain concrete samples: from 8.14 to 3.14%, 41.19 to 36.17%, and 70.06 to 66.35%, after exposure to 400, 600 and 800 °C, respectively. In another work [39], the effects of various heating regimes on the split-tensile strength of HSCs (w/c = 0.29), containing 0 and 3 wt. % of NS, were analyzed. The samples were exposed to temperatures from 200 to 800 °C, with 0 min, 1 h and 2 h of constant heating at the desired temperature. It was observed that the split-tensile strength of NS-modified specimens increased up to temperatures of 400 °C (in all heating regimes), while plain concrete exhibited split tensile strength improvement only up to 200 °C. Afterwards, the strength of the plain concrete was lower than that of the unheated specimen. After exposure to 400 °C for 2 h, the split-tensile strength of NS-modified HSC specimens was higher, by 13%, than that of the unheated specimens, while the control HSC lost approximately 30% of its strength. The authors linked these findings with the formation of a high-density volume fraction of C–S–H (which was the result of increased C–S–H silicate chain length), which thus improved the stability of C–S–H. After exposure to 600 °C and 800 °C, split-tensile strength dropped significantly and the differences between the control and NS-modified concrete decreased. After exposure to 800 °C, the differences between samples were <10%. In work undertaken by Yan et al. [51], the effect of 2 wt. % of NS admixture on the split-tensile strength of SFRC, after exposure to 200, 400, 600, and 800 °C, was analyzed (Figure 3). Up to 400 °C, an increment in the split-tensile strength of concrete was observed, after which a loss of strength was reported. It was observed that up to 600 °C, NS-modified SFRC specimens exhibited higher strengths than the control SFRC specimen. The highest split-tensile strength values of concrete specimens were reported after exposure to 400 °C, with the NS-modified SFRC and control SFRC exhibiting strengths of 5.0 MPa and 4.5 MPa, respectively. After exposure to 600 °C, NS-modified SFRC exhibited a strength similar to that of the unheated sample, while the control SFRC had a lower strength than that of the corresponding unheated sample. After 800 °C, the split-tensile strength of all the concretes was similar.

In a subsequent work [50], the effect of 2 wt. % NS admixture on the axial tensile strength of SFRC, after exposure to 200, 400, 600, and 800 °C, was analyzed. The authors reported significant strength improvement in NS-modified SFRC specimens, in unheated states, as compared to a control SFRC. In all samples tested up to 400 °C, strength improvements were reported. However, after thermal exposure, the strength ratio between the NS-modified SFRC and the control SFRC decreased. Nevertheless, NS-modified specimens were superior to control SFRC specimens, up to a temperature of 800 °C.

From the references cited above, it is safe to conclude that the inclusion of NS in cement-based composites is beneficial for improving the tensile strength of composites working under elevated temperature conditions. A significant improvement of tensile strength is observed when exposed to temperatures up to 400 °C, while further increments of temperature result in a decrease in the ratio between NS-modified and plain specimens. In general, it can be stated that NS reduces the tensile strength loss resulting from exposure to high temperatures, with different grades of change, depending on the temperature applied.

#### 2.2.3. Compressive Strength of Cement Pastes

**Figure 4:**
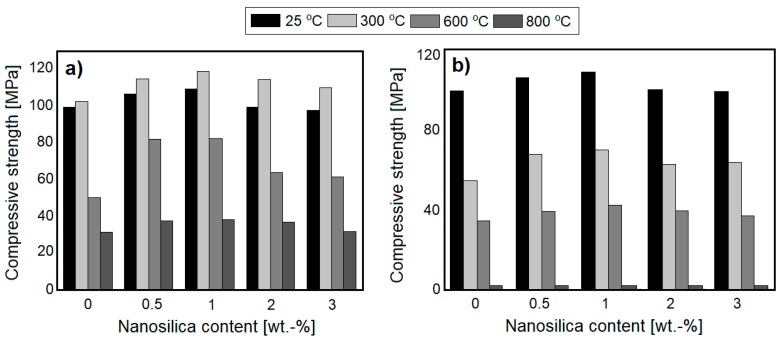
Compressive strength of cement pastes containing NS, after heating and cooling: (**a**) gradually in air, (**b**) suddenly in water. Reproduced with permission from [41]. Copyright Springer Nature, 2018.

**Figure 5:**
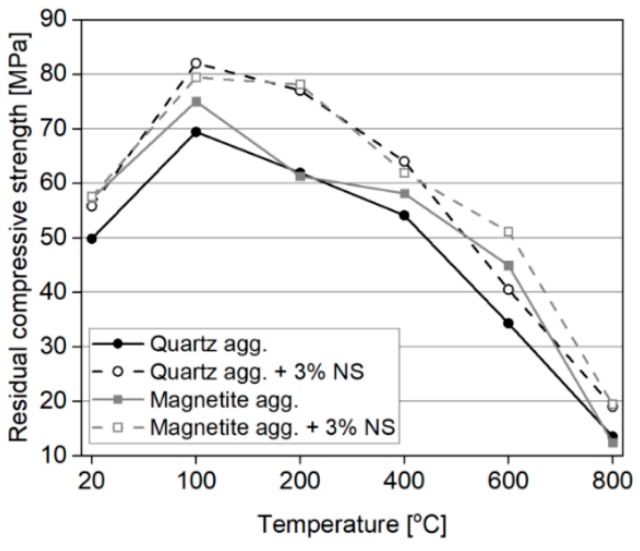
Comparison of residual compressive strengths of cement mortars containing quartz and magnetite aggregate, with and without NS. Reproduced with permission from [28]. Copyright Elsevier, 2017.

The effect of NS on the compressive strength of cement pastes exposed to elevated temperatures has been reported in several works [40,41,42,52]. Heikal et al. [52] analyzed the effects of 1–6 wt. % of NS as a cement replacement in OPC pastes and pastes with GGBS, with specimens heated up to 1000 °C. Generally, it was observed that incorporation of NS in cement pastes helped to retain the strength of cement pastes up to 1000 °C, although a much more beneficial effect of NS was observed in cement pastes containing GGBS and 4 wt. % of NS. In another work [40], the effects of NS in the amounts of 1 and 4 wt. % and GGBS from 0, 25, 50, and 65 wt. %, as a cement replacement, on the thermal resistance of cement pastes with or without superplasticizer, exposed to temperatures up to 950 °C, were analyzed. The study showed that the incorporation of superplasticizer, together with NS, has the most beneficial effect in improving the thermal resistance of cement pastes. In the case of OPC paste, 1 wt. % of NS was established as the most beneficial amount for improving heat resistance up to 400 °C. In the case of GGBS-modified pastes, a positive effect of NS was observed up to 650 °C. Lim et al. [42] analyzed the effects of 5 wt. % of NS addition, on the thermal resistance of cement pastes exposed to 100–500 °C, with two different cooling regimes: cooling down to room temperature and prolonged heat treatment at 50 °C for 3 days. The results were compared with plain OPC specimens: after cooling down from all heating temperatures, NS-modified specimens exhibited higher compressive strength, by 7 to 20%, as compared to control OPC specimens. The most noticeable difference in compressive strength between samples was observed after exposure to temperatures of 400 °C and 500 °C, with the values being 20% and 17%, respectively. When samples were cooled after prolonged heat treatment regimes, a significant change in performance between NS-modified and plain OPC specimens was observed, after exposure to 500 °C. The study showed that plain OPC samples broke down completely and lost their strength, while samples containing NS retained almost 80% of their initial strength. El-Gamal et al. [41] have evaluated the effects of 0.5–3 wt. % NS admixture on the properties of OPC cement pastes, exposed to temperatures of 300, 600, and 800 °C (Figure 4). Two different cooling regimes, gradually in air and water quenching, were evaluated. In the case of the first testing regime (Figure 4a), all of the samples tested exhibited a slight strength increment after exposure to 300 °C, although the samples containing 0.5, 1, 2, and 3 wt. % exhibited strength improvements by 8, 9, 14, and 12% (relative to the strength of unheated samples), while the control OPC exhibited only a 3% improvement in strength. After exposure to 600 °C, all the specimens tested, exhibited strength losses, although samples containing 0.5, 1, 2, and 3 wt. % of NS retained 77, 75, 64, and 63% of their initial strength (respectively); for the control sample only 51% of the initial strength was retained. After exposure to 800 °C, all the samples tested exhibited only 31–35% of their initial strength, with no significant differences between samples being observed. In the case of a rapid cooling regime through water quenching (Figure 5b), the drop in strength was observed at all the testing temperatures, while at a temperature of 800 °C the samples lost their strength and broke down. Nevertheless, samples containing NS retained a higher compressive strength after exposure to 300 °C and 600 °C, with the authors concluding that the optimal NS addition value is 1 wt. %.

#### 2.2.4. Compressive Strength of Cement Mortar

The effects of NS on the thermal resistance of cement mortars has also been analyzed in several studies [28,43,44,45]. In a study undertaken by Horszczaruk et al. [28], the effect of NS as an admixture (from 1 to 5 wt. %) on the compressive strength of cement mortars containing either quartz, magnetite or barite aggregate and exposed to temperatures up to 800 °C, was determined. The study showed that there was an optimum amount of NS (3 wt. %), which was most beneficial for improving the compressive strength of cement mortars containing quartz and magnetite aggregate. The beneficial effect of NS was mainly noticeable up to a temperature of 400 °C. After this point, the effect of NS on compressive strength was less significant or negligible. Examples of the effects of NS on the compressive strength of cement mortars with quartz and magnetite aggregates have been plotted in Figure 5. In the case of cement mortars containing barite aggregate, the inclusion of NS contributed to increased cracking potential (as compared to pristine samples), with the compressive strength of specimens after heating thus being lower. However, this phenomenon occurred due to the high water absorption of the barite aggregate, which resulted in undesired compaction of the cement matrix. As such, the effect of NS is dependent on the type of aggregate used.

Ibrahim et al. [45] have analyzed the effects of temperature on the compressive strengths of OPC mortars containing PP fibers and NS in the amounts of 0, 2.5, 5, and 7.5 wt. %. Their samples were cured for 3, 7, and 28 days and then exposed to temperatures of 400 and 700 °C. The compressive strengths of the cement mortars increased after exposure to 400 °C, but higher strength increments were reported for specimens containing NS. Strength gains were much more noticeable in the early stages of curing, as a result of accelerated pozzolanic reactions induced by heating in the presence of NS, which led to the production of higher amounts of C–S–H phase than in plain mortar. After exposure to 700 °C, a remarkable reduction in compressive strength was observed in all specimens, with the effect of NS being negligible. In another study [44], the authors detected a synergistic effect of FA and NS in the production of heat-resistant mortar. High-volume fly ash cement mortars (w/b = 0.4), containing 25, 35, and 45 wt. % of cement replaced with FA and 2.5, 5, and 7.5 wt. % of NS, were produced. The specimens were exposed to 400 and 700 °C, after 3, 7, and 28 days of curing. The authors reported that after exposure to 400 °C, all their specimens exhibited an increase in compressive strength, although improvements were more noticeable for specimens containing NS. After exposure to 700 °C, the samples exhibited significant strength loss, but an optimal combination of FA and NS (37.5 wt. % and 7.5 wt. %, respectively), enabled the production of mortar that exhibited compressive strength comparable to that of the unheated specimen.

Results contrary to those presented above, have been reported by Maheswaran et al. [43]. In this study, the compressive strengths of cement mortars (cured for 3, 7, and 28 days) and containing 20 wt. % of cement replaced with lime sludge and NS (1.5 and 3 wt. %) or SF (3 and 6 wt. %), after exposure to 500 and 800 °C, were determined. Generally, after exposure to 500 °C (regardless of the time of curing), specimens containing NS exhibited more strength loss than the control sample. The only exception was mortar containing 1.5 wt. % of NS, cured for 3 days. In addition, samples containing NS showed worse performance than those containing SF. After exposure to 800 °C, specimens containing NS exhibited worse mechanical performance than the plain control sample. However, their performance was better than that of SF-incorporated specimens, which exhibited spalling and lost their strength, while NS-incorporated samples exhibited higher strength and were not damaged.

#### 2.2.5. Compressive Strength of Concrete

**Figure 6:**
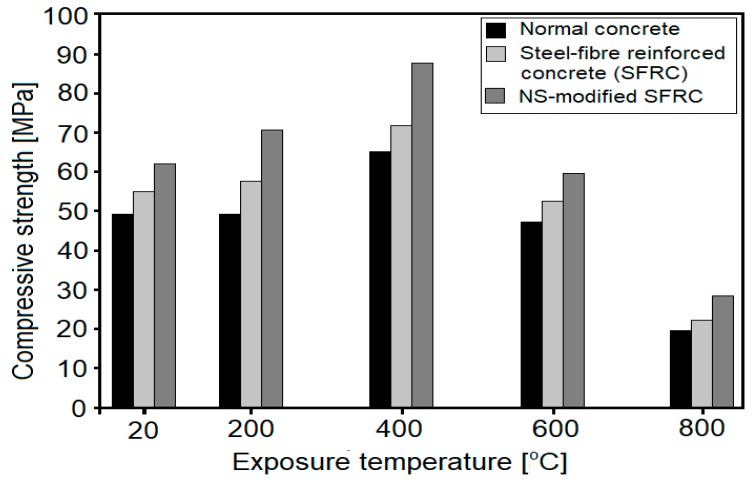
Compressive strength of normal concrete, SFRC and NS-modified SFRC, as a function of temperature. Redrawn from [51].

The effect of NS on the compressive strength of concretes, after thermal exposure, have also been analyzed in several studies [39,46,47,48,49,51]. Kumar et al. [39] analyzed the effect of thermal exposure (from 200 to 800 °C) of HSCs (w/c = 0.29), containing 0 and 3 wt. % of NS, on compressive strength. The samples were heated under three different regimes, with varying times of maintaining the desired temperature (0 min, 1 h and 2 h). The authors observed that in NS-incorporated concretes, compressive strength increased up to 400 °C (in all heating regimes), while plain concrete exhibited compressive strength improvements only up to 200 °C. Beyond 200 °C, the strength of the reference concrete was lower than that of the unheated specimen. It is worth noting, that the relative compressive strength of NS-modified HSC, after exposure to 400 °C (with a 2 h heating regime), increased by 40%. This effect was attributed to the improved stability of the C–S–H phase and higher volume of high-density C–S–H. Further thermal exposure (600 and 800 °C), led to a gradual strength decrement of the specimens, with NS-modified samples exhibiting only slightly higher relative compressive strength than plain HSC (with a difference less than 10%). Rathi and Modhera [48] analyzed the effects of HSCs with cement replacements: from 10 wt. % to 30 wt. % of FA (increments of 5 wt. %), or from 1 to 5 wt. % of NS (increment steps of 1 wt. %). For comparison, plain concretes without NS or FA were prepared. The concrete samples were exposed to temperatures of up to 800 °C. The study showed that the incorporation of NS in the amount of 3 wt. % was beneficial in improving the thermal resistance of concretes, up to 800 °C. In the presence of NS, a compressive strength increment was observed, up to 400 °C. Moreover, the best performance among all the concretes tested, was exhibited by concrete based on a mixture of FA (20 wt. %) and 3 wt. % of NS. Yan et al. [51] have characterized the effects of 2 wt. % of NS admixture on the compressive strength of SFRC exposed to 200, 400, 600 and 800 °C. The results were compared with plain SFRC and normal concrete, showing that the presence of NS remarkably increases the compressive strength of concrete (by 41%), up to 400 °C, as compared to reference concrete (Figure 6). Beyond 400 °C, strength decreased and at 600 °C the compressive strength was comparable to that of the unheated specimens. Furthermore, all the samples lost more than half of their initial strength, after exposure to 800 °C.

Bastami et al. [46] characterized the effects of replacing SF with NS, in two types of HSCs (w/b = 0.25), containing 30 kg/m^3^ and 60 kg/m^3^ of SF and exposed to temperatures of 400, 600, and 800 °C. SF was replaced with NS in the amounts of 1.5, 3, and 4.5% by a mass of cement (1.41, 2.83, and 4.2 wt. % of binder mass, respectively). The study showed that in both types of concrete, residual compressive strength increased with NS content. For example, the strength loss of plain concrete (SF = 30 kg/m^3^), after exposure to 400, 600, and 800 °C was 15.3%, 48.4%, and 73.3%, while the corresponding HSC with 4.5 wt. % of NS lost its strength by 7.1%, 40.0%, and 67.9%, respectively. Sherif [49] analyzed the thermal resistance of two types of concretes, with different cement amounts of 350 and 450 kg/m^3^ (fixed w/c = 0.25) and an addition of SF by 15 wt. % and of NS by 1–5 wt. %. Their samples were exposed to temperatures up to 600 °C. A slight compressive strength improvement was observed after exposure to 200 °C; however, no differences between specimens were reported. After exposure to 400 and 600 °C, the samples gradually lost their strength, however lower strength loss was reported for concretes containing NS. Also, with an increment of NS content, the strength loss was reduced. After exposure to 400 and 600 °C, both types of plain concrete (with cement contents of 350 and 450 kg/m^3^) exhibited 78.0%, 40.4% and 70.0%, 36.2% of their initial strength, respectively, while NS-incorporated concrete (5 wt. %) exhibited 86.0%, 49.0%, 77.0%, and 44.5% (respectively) of their initial strength.

Contradictory results have been presented by Shah et al. [47]. In their study, three HSC mixes (w/b 0.25–0.3) containing 5 wt. % of NS, 10 wt. % of SF or a mixture of 5 wt. % of NS and 5wt. % of SF, were prepared. The specimens were exposed to temperatures of 200, 500, and 800 °C. At the first heating temperature (200 °C), a beneficial effect of NS was observed, with concrete containing 5 wt. % of NS exhibiting the highest strength improvement (by 10%). However, after exposure to 500 and 800 °C, all the samples gradually lost their strength, with the highest strength loss being observed for samples containing NS. After exposure to 800 °C, NS-modified samples lost 79% of their initial strength, while specimens containing SF exhibited a strength loss of 49%. Incorporation of an NS/SF mixture resulted in a 71% strength loss. The authors attributed this phenomenon to an excessive build-up of vapor pressure, which led to extensive cracking in NS-incorporated samples. Moreover, the authors reported a significant reduction of workability and problems with homogenous mixing, when NS was present in the mix. This could have been the factor resulting in the negative impact of NS present in the mix.

Based on the above-mentioned research, it is safe to conclude that the incorporation of NS has a significant impact on the improvement of compressive strength, after exposure to elevated temperatures, regardless of the type of composite (i.e., paste, mortar or concrete). The inclusion of NS has a beneficial effect on compressive strength retention, even up to a temperature of 800 °C. However, in most of the works cited, NS-incorporated specimens exhibited a remarkable improvement in compressive strength up to 400 °C, after which the beneficial effect diminished gradually.

### 2.3. Microcracking and Spalling

As mentioned in the introduction, incorporation of selected SCMs, such as SF, represents a significant threat related to the risk of increased cracking and spalling. Spalling is more likely in HSCs (with lower water to cement ratio). Due to the filler effect, pozzolanic activity, and ultra-fine particle size, the incorporation of SF results in concrete compaction and the refinement of the pore structure, thus resulting in a cement matrix with low permeability. Heating of the concrete results in a build-up of internal pressure, leading to extensive cracking. However, assuming NS action based on previous findings regarding SF might be misleading, due to the different reactivity of NS, as well as the lower amount of NS incorporated (usually lower than 5 wt. %).

Generally, in cement-based composites with standard w/c ratios, it is assumed that NS has a beneficial effect in decreasing the number of surface cracks in cement-based composites. Due to an increase in the amount of high-density C–S–H phase and the higher thermal resistance of the C–S–H phase in a lower temperature range (up to 300–400 °C), fewer cracks are observed in the cement matrix. After exposure to a higher temperature range (above 400–450 °C), the amount of surface cracks in NS-modified cement-based composites has also been observed to be lower and that microcracks are narrower and shorter. This effect is attributable to the fact that NS decreases the amount of CH in the cement paste, because of its reaction with CH, resulting in the formation of an additional amount of C–S–H. At a temperature of 400–450 °C, decomposition of free Ca(OH)_2_ to CaO occurs. During cooling and exposure to moisture, the dehydrated CH reforms rapidly, accompanied by a noticeable volume increment, resulting in the further development of microcracks. However, due to a lower amount of initial CH in NS-incorporated samples (before heating), fewer microcracks are observed after heating in temperatures >400 °C [28].

Bastami et al. [46] have analyzed the effect of NS, (as a SF replacement, in the amounts of 1.5, 3 and 4.5 wt. %) on spalling, in two types of HSC (w/b = 0.25), containing fixed amounts of silica fume; i.e., 30 kg/m^3^ and 60 kg/m^3^. The concretes were exposed to 400, 600, and 800 °C, with the authors reporting that the effect of NS on spalling was not easy to judge, but that a few general conclusions could be drawn: up to 400 °C, no spalling was observed and mass loss for all mixtures containing NS was lower than that of the reference sample. After exposure to 600 °C, spalling was observed for all the samples tested. In the case of HSC with a fixed SF (or SF + NS) amount of up to 30 kg/m^3^, the presence of NS decreased spalling. Samples containing NS exhibited a lower mass loss, as compared to plain samples. However, the presence of NS in HSC with a fixed SF (or SF + NS) amount of up to 60 kg/m^3^, resulted in significantly increased spalling. For instance, the reference mixture exhibited a mass loss of 9.3%, while samples containing 1.5, 3 and 4.5 wt. % of NS exhibited mass losses of 22.9, 16.4, and 11.0%, respectively. After exposure to 800 °C, a similar trend in the spalling of the samples was observed. The first type of concrete (with a lower SF/SF + NS content), exhibited lower spalling (lower mass loss), while the concrete with 60 kg/m^3^ of SCMs exhibited higher spalling (higher mass loss), when NS was present in the mix. Shah et al. [47] have studied the spalling of three HSCs (w/b = 0.25–0.3), containing 5 wt. % of NS, 10 wt. % of SF or a mixture of 5 wt. % of NS and 5 wt. % SF, exposed to 200, 500, and 800 °C. After exposure to 200 °C, no visible cracks on the surface of the specimens were observed, with the first spalling and cracking being observed only after exposure to 500 °C. It was noted that samples containing SF exhibited some fine surface cracks, while the samples containing NS exhibited significant cracks. However, the highest cracking and spalling was observed in samples containing a mixture of NS and SF. After exposure to 800 °C, the authors reported that less cracks were observed in specimens containing SF, while extensive cracking (with cracks going deep into the cross-section) was observed for samples incorporated with NS. Spalling occurred in samples containing a mixture of NS and SF. The authors attributed this phenomenon to the extra dense microstructure produced by the incorporation of a combination of SF and NS. 

Horszczaruk et al. [28] analyzed the effects of the presence of NS on cement mortars containing various aggregates (quartz, magnetite and barite). Generally, a positive effect, in terms of a reduction in crack width and length was observed, but in the case of the barite aggregate, undesirable spalling and cracking occurred. This was attributed to the high water absorption of the barite aggregate itself, resulting in the accumulation of water in the aggregate and an undesired compaction of the cement matrix. When NS was incorporated in the mix, a dense microstructure was produced. Due to the high thermal conductivity of the barite aggregate, a fast diffusion process of absorbed water associated with the aggregate, occurred. When the specimens were heated, the denser microstructure of NS-modified mixes resulted in more extensive cracking, in comparison to the plain specimens. Maheswaran et al. [43], have analyzed the effects of ternary blended OPC, containing lime sludge and SF or NS, exposed to 500 and 800 °C. No spalling in cement mortars was observed up to 500 °C, while beyond 800 °C, spalling was observed in samples containing SF. On the other hand, the NS-modified samples did not exhibit a spalling effect, as a result of the apparent pore-filling effect of NS and enhanced particle packing in the mortar.

From the references cited above, it can be seen that most of studies have reported beneficial effects of NS regarding crack reduction in cement-based composites, especially in the case of composites with a higher w/c content. Compared to silica fume, the incorporation of NS reduces the risk of cracking in cement-based materials with higher w/c ratios, after exposure to high temperatures. However, the effect of NS on spalling in composites with denser matrices, such as HSCs, is still controversial and requires further investigation. 

## 3. The Effect of Carbon-based Nanomaterials on the Thermal Resistance of Cement-Based Composites

Along with NS, particular research interest has also arisen in regard to the incorporation of carbon-based nanomaterials in cementitious composites: mainly carbon nanotubes (CNTs), carbon nanofibers (CNF), graphene oxide (GO), and graphene nanoplatelets (GNPs) [30]. Due to their reinforcing ability (high tensile strength and high toughness), the incorporation of CNTs and CNFs, even in very small amounts (usually less than 0.5 wt. %), significantly improves mechanical performance, fracture characteristics, and the durability of cementitious composites. CNTs create bridges between nano- and micro-cracks in the binder, thus increasing tensile strength and limiting further crack propagation [23,53,54]. In addition, due to their remarkable mechanical and electrical properties, CNTs are ideal for manufacturing self-sensing cement-based composites; whereby the cement-based composites act as sensors able to detect their own state of strain or stress, as reflected in changes to their electrical properties [55,56]. GO has also been introduced as an excellent reinforcement for cementitious composites, due to its easier method of production (as compared to CNTs) and higher solubility in aqueous cement matrices. In addition, graphene oxide has a higher surface area than carbon nanotubes, and its surface is covered with higher amounts of functional groups. Therefore, GO sheets exhibit higher reactivity (compared to CNTs), and thus increase the nucleation area for the C–S–H gel [30,57,58]. The greater number of functional groups dispersed all across the surface area, make this material ideal for incorporation in cementitious composites, as cement matrix “nano-reinforcers”.

Nevertheless, interest related to the influence of carbon-based nanomaterials on the performance of cement-based composites, under various thermal conditions, is relatively limited [59,60,61,62]. However, the available data shows very promising results regarding the incorporation of carbon-based nanomaterials, to produce heat-resistant cementitious composites. The studies available, related to the effects of carbon nanomaterials, are summarized in Table 3.

### 3.1. The Effect of Multi-Walled Carbon Nanotubes (MWCNTs)

**Figure 7:**
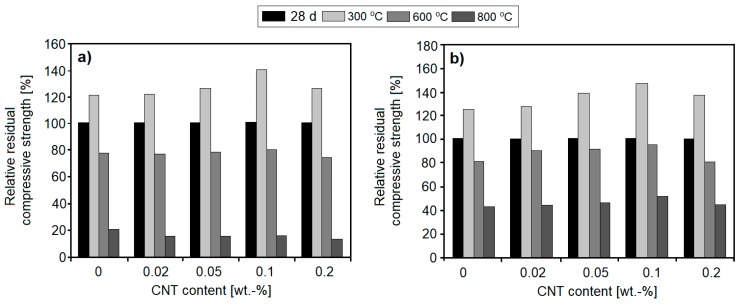
The effect of carbon nanotubes (CNT) content on the relative residual compressive strength of cement pastes containing 100 wt. % of ordinary Portland cement (OPC) (**a**) and 70 wt. % of OPC and 30 wt. % of Homra (**b**) after firing. Reproduced with permission from [59]. Copyright Elsevier, 2015.

Amin et al. [59] have analyzed the effects of MWCNTs, in the amounts of 0.02, 0.05, 0.1, and 0.2 wt. %, on the thermal resistance of OPC pastes and cement pastes containing up to 30 wt. % clay brick waste (Homra) cement replacement. The cement pastes (w/b = 0.3) were exposed to temperatures of up to 800 °C (Figure 2). The results showed that an optimal amount of MWCNTs (established as 0.1 wt. %) in combination with clay brick waste resulted in a marked increase of residual compressive strength, in cement pastes exposed to elevated temperatures, even up to 800 °C for certain paste compositions (Figure 7b). However, in the case of OPC pastes, the beneficial effect of MWCNTs was only visible up to 300 °C (Figure 7a). X-ray powder diffraction (XRD), differential scanning calorimetry (DSC), and scanning electron microscope (SEM) analysis showed that MWCNTs do not affect the rate of hydration reactions. The positive effect of MWCNTs can most likely be attributed to their pore-filling effect, as well as to their bridging ability between hydrates and across cracks.

**Figure 8:**
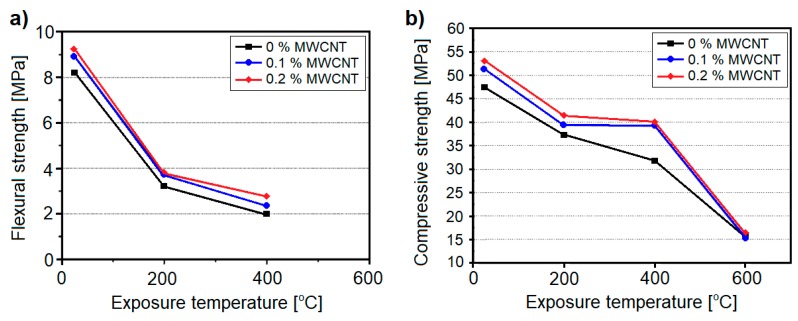
The effect of hydroxylated Multi-Walled Carbon Nanotubes (MWCNTs) on the flexural (**a**) and compressive (**b**) strengths of cement pastes exposed to elevated temperatures. Reproduced with permission from [63]. Copyright Elsevier, 2017.

Similar conclusions have been drawn by Zhang et al. [63]. In their work, the effect of 0.1 and 0.2 wt. % of hydroxylated MWCNTs on the microstructure and mechanical performance (flexural strength and compressive strength) of cement pastes (w/c = 0.4), exposed to temperatures up to 600 °C, was determined. XRD results showed that the inclusion of MWCNTs does not affect the hydration kinetics of cement at room temperature, while at elevated temperatures, MWCNTs hindered further hydration. After exposure to 600 °C, no influence of the MWCNTs on the decomposition of hydration products was observed. SEM analysis revealed that up to a temperature of 400 °C, CNT exhibited a bridging effect for the pores and cracks in the cement matrix, while after exposure to 600 °C, the MWCNTs were mostly spalled with the matrix on the walls of the pores and cracks. The positive effects of the MWCNTs were reflected both in the flexural and the compressive strength of cement pastes (Figure 8). Up to 200 °C, strength enhancement was attributable to the continued existence of the bridging of CNT in the gaps and pores. However, the beneficial effects of the MWCNTs on thermal resistance were most pronounced after exposure to 400 °C. It is worth noting that MWCNT-incorporated samples, exhibited similar compressive strengths after exposure to 200 °C and 400 °C, while plain cement paste exhibited gradual strength loss after exposure to these temperatures (Figure 8b). Therefore, it was carbon nanotubes that contributed to the prevention of the loss of compressive strength, after exposure to 400 °C. The authors attributed this phenomena to the potential ability of CNTs to work as channels for the release of autoclaving steam, thus preventing damage from high-pressure steam. After exposure to 600 °C, the MWCNTs lost their bridging ability, and the samples exhibited a similar compressive strength. The optimal value of MWCNTs in the paste was estimated at 0.2 wt. %.

**Figure 9:**
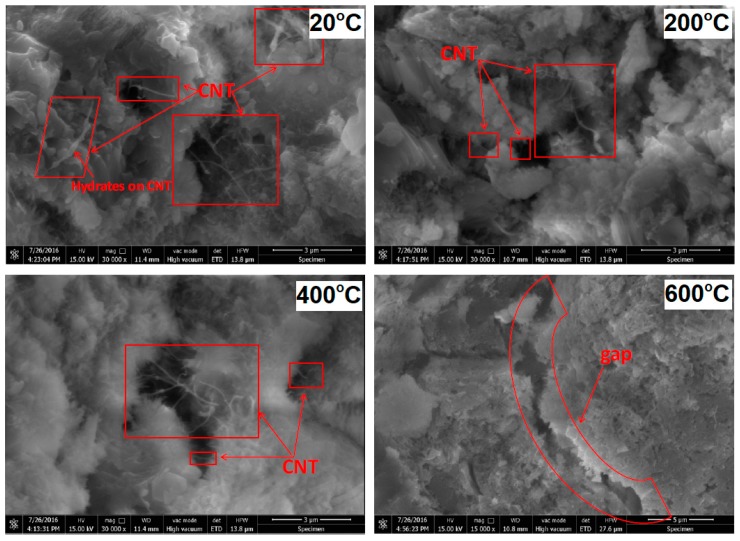
Scanning electron microscope (SEM) micrographs showing the bridging effect of MWCNTs in a cement matrix before (20 °C) and after exposure to elevated temperatures of 200, 400, and 600 °C. Reproduced with permission from [63]. Copyright Elsevier, 2017.

Moreover, extensive SEM analysis (Figure 9) showed that up to a temperature of 400 °C, the bridging phenomenon of CNT in the cement matrix can be observed, while at a temperature of 600 °C carbon nanotubes are not discernible.

### 3.2. The Effect of Carbon Nanospheres

Another approach for incorporating carbon-based materials to improve the thermal resistance of cement-based composites, has been proposed by Han et al. [61]. In this study, the authors proposed a novel multiscale reinforcement structure, built from the fast growth of carbon nanospheres (CNSs) on the surface of carbon fibers (CFs). The fiber surface was covered with a uniform CNS layer, with an average thickness of 85 nm. Afterwards, cement pastes (w/c = 0.33) containing 0.55 vol. % of fiber were prepared and subjected to temperatures of 200, 400 and 600 °C. It was observed that as the temperature increased, the size of the cracks in the cement pastes increased. Crack sizes were smallest in the samples containing CNS-modified fibers. The study showed that, cement pastes containing CNS-modified CFs, exhibited better resistance to the thermal degradation (up to 600 °C) resulting from exposure to elevated temperatures, with superior relative residual strength. The authors attributed the positive effect of CNS on the surface of CF, to two main phenomena: Firstly, to the improved interlocking between CNS-modified CFs and the cement matrix, as compared to pristine CFs; and secondly, to the improved thermal resistance of CFs, resulting from a thin CNS layer that oxidizes first, thus forming microchannels which enable vapor tension in the capillaries to be more easily alleviated and released. 

### 3.3. The Effect of Graphene Oxide

**Figure 10:**
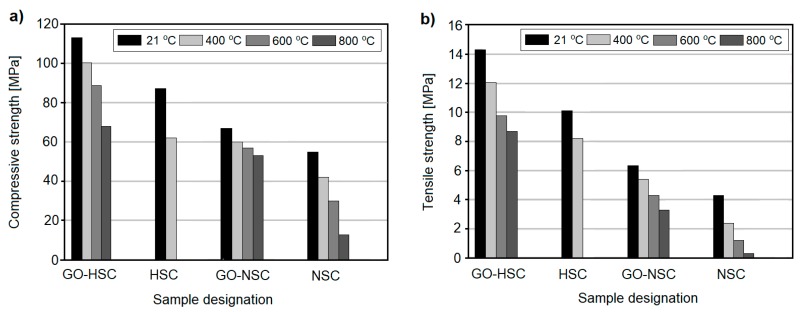
Compressive strength (**a**) and tensile strength (**b**) test results of graphene oxide (GO)-modified high strength concrete (GO-HSC), high strength concrete (HSC), GO-modified normal strength concrete (GO-NSC) and normal strength concrete (NSC) after exposure to elevated temperature. Redrawn from [62].

Mohammed et al. [62] incorporated graphene oxide in normal strength concrete (w/c = 0.45) and HSC (w/c = 0.30), exposing them to temperatures up to 800 °C. In the case of normal strength concrete, the compressive strength loss (Figure 10a) of GO-modified concretes was noticeably lower than that of plain concrete, especially after exposure to 400 and 600 °C (Figure 10a). Plain, normal strength concrete exhibited a gradual strength loss after exposure to 400, 600, and 800 °C, while GO-modified concrete exhibited only a slight strength reduction. In the case of HSC, incorporation of GO in the mix was even more efficient than in the case of normal strength concrete. It is known that HSCs are more prone to spalling and degradation under elevated temperature conditions, but the authors reported that the spalling resistance of GO-incorporated HSC increased, such that samples were not damaged, even up to 800 °C and that strength loss was gradual. However, in the case of plain HSC, specimens were destroyed above 400 °C. Similar trends were observed in the case of the tensile strength of concretes, although in this case, the degree of strength degradation was higher (Figure 10b).

The authors attributed the beneficial effect of GO on mechanical properties to its ability of minimizing temperature increase in the specimen and thus limiting the effects of temperature exposure, as well as reducing cracks in the concrete. The authors reported that the incorporation of GO contributed to alternation in the porosity of the cement matrix. The number of large pores (from 0.4 μm to 10 μm) shifted to a number of small pores (<0.3 μm), which contributed to an increment in the amount of effective gel pores. As such, porous structures with nano- and micro-scale channels were produced, enabling the release of vapor pressure, helping to prevent extensive spalling in samples, which was reflected in the mechanical properties of the concretes as well as in the decreased amount of cracks [62]. 

### 3.4. The Effect of Graphene Sulphonate Nanosheets

Chu et al. [60] analyzed the effect of graphene sulfonate nanosheets (GSNSs), in the amount of 0.1 wt. %, on the properties of sacrificial concretes (w/b = 0.33) exposed to temperatures of up to 1000 °C. Extensive SEM analysis and MIP tests showed, that the inclusion of GSNSs exhibited reinforcing and toughening effects on the microstructures of the concretes tested, which was reflected in a reduction of concrete porosity. The total porosity of concrete modified with GSNSs was reduced by 3.0–7.0% (depending on the heating temperature), as compared to plain concrete samples. Moreover, the thermal gradient in the concrete modified with GSNSs decreased and as such, the thermal damage rate dropped. The positive effect of GSNSs on microstructure translated in to an improvement in split-tensile and compressive strength. The authors reported, that relative residual compressive strength, as well as the splitting tensile strength of GSNS modified concretes, was higher than that of plain concrete, at each tested temperature.

## 4. The Effect of Nanoclays and Calcinated Nanoclay on the Thermal Resistance of Cement-Based Composites

Nanoclays (NCs) originate from naturally occurring clays. These clay particles are hydrous silicates and can generally be described as fine-grained particles organized in sheet-like structures stacked on top of each other [74]. Based on their chemical composition and morphology, NCs have been classified into various groups, including montmorillonite, bentonite, kaolinite, hectorite, and halloysite [31]. The incorporation of NCs has been gathering increasing attention in many fields of engineering, which is attributable to their availability, environmental friendliness, and low cost, as compared to other manufactured nanomaterials. The beneficial effects of NC admixtures on the properties of cement-based composites has been widely reported. Due to their large surface area to volume ratio, NC particles can facilitate chemical reactions. In the presence of silica and aluminum, NC nanoparticles can exhibit a nucleation effect and high pozzolanic activity (as in case of nanosilica), as well as a nano-filling effect, which leads to an increase in the overall performance of concrete [24]. Previous studies have shown that the inclusion of NC contributes to the formation of ill-crystalline CH and the formation of a C–S–H phase [75]. Chang et al. [76] have reported that the incorporation of nano-montmorillonite results in higher CH consumption and higher production of C–S–H. In addition, the study showed that NCs are non-combustible materials and as such, various efforts to incorporate them as flame and fire protective admixtures in a vast range of materials, including polymers, have been undertaken. [74]. Based on these observations, attempts to incorporate NCs as cement admixtures for improving the thermal resistance of cement-based composites, has also gathered researchers’ attention. Table 3 summarizes the studies related to the thermal resistance of NC-modified cement-based composites.

**Figure 11:**
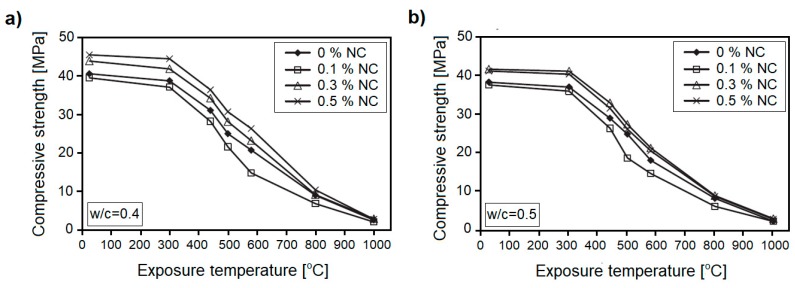
Compressive strength of nanoclay-modified concrete with w/c = 0.4 (**a**) and w/c = 0.5 (**b**) after thermal exposure. Reproduced with permission from [67]. Copyright Elsevier, 2017.

Wang [67] have analyzed the effects of cement replacement, by 0.1, 0.3, and 0.5 wt. %, on the thermal resistance of two types of concrete, with w/c = 0.4 and w/c = 0.5. In their study, compressive strength and the thermal conductivity coefficient, under different temperature conditions (up to 1000 °C), were analyzed. It was shown that in both types of concrete, when 0.1 wt. % of NC was incorporated in the concrete, compressive strength loss was higher than that of plain concrete, during heating (Figure 11). However, when the amount of NC was increased to 0.3 wt. % and 0.5 wt. %, the highest compressive strength increase, during heating and relative to ordinary concrete, was observed. In addition, the authors reported that the thermal conductivity coefficient of cement paste with 0.1 wt. % NC dropped, while the replacement of cement with 0.3 wt. % and 0.5 wt. % NC, increased the thermal conductivity coefficient of paste.

**Figure 12:**
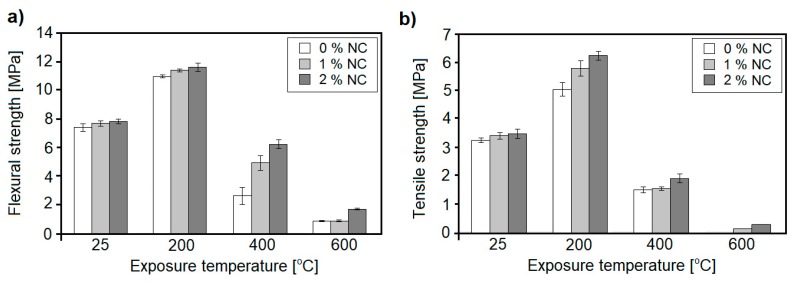
Flexural strength (**a**) and tensile strength (**b**) of cement mortars, containing NC, exposed to elevated temperature. Reproduced with permission from [64]. Copyright Elsevier, 2018.

In a study undertaken by Irshidat et al. [64], the effect of hydrophilic montmorillonite, on the thermal resistance of cement mortars (w/b = 0.55) containing a NC cement replacement rate in the amount of 1 and 2 wt. %, exposed to 200, 400, and 600 °C, was examined. The authors determined the effect of NC on flexural strength, tensile strength, and compressive strength and supported their study with XRD, DSC and TGA analysis. The effect of NC on the compressive strength of cement mortars was relatively limited and only a slight compressive strength improvement during heating was observed, when 2 wt. % NC was incorporated in the mix. The effect of NC was much more pronounced regarding flexural and tensile strength (Figure 12). After exposure to elevated temperatures, NC-incorporated samples exhibited higher flexural strength under all heating temperatures (Figure 12a). After exposure to 200 °C, samples containing NC exhibited only a slightly higher flexural strength than plain mortar, however, when the temperature increased to 400 °C, a significant difference between the flexural strength of unmodified and modified mortars was observed. Samples containing 1 and 2 wt. % NC exhibited 88% and 138% higher flexural strength than plain cement mortar. Further exposure to a temperature of 600 °C caused a dramatic strength loss in cement mortars, with the specimen containing 2 wt. % of NC exhibiting the highest flexural strength value. The beneficial effect of NC was also reflected in the tensile strength of mortars (Figure 12b).

After exposure to 200 °C, NC-modified samples exhibited a higher increment in tensile strength, as compared to the plain mortar. After exposure to 400 °C, the strength of the specimens declined and the difference between samples was reduced; however, the highest strength was represented by samples containing 2 wt. % of NC. Exposure to 600 °C caused a total loss of strength in plain cement mortar, while NC-incorporated samples still exhibited some strength. XRD analysis confirmed that in the presence of 2 wt. % nanoclay, the consumption of CH reached its optimum value at 200 °C, whereas the additional formation of a C–S–H phase in the presence of NC reached its optimum level at 400 °C. In addition, SEM analysis showed that the presence of NC decreased the density and width of microcracks in the cement mortar, after heating, as compared to the plain mortar. 

The beneficial effects of NC on the compressive, flexural and split-tensile strengths of concretes, has also been reported by Ho et al. [66]. In their study, NC was added to concrete in 0.1, 0.3, and 0.5 wt. %, with concrete specimens being exposed to temperatures of 400, 600, and 800 °C. It was found that the exposure of concretes with NC, up to a temperature of 400 °C, barely affected their compressive strength (loss by 1–2 MPa), while the control concrete lost 8.5 MPa of its initial strength. Further exposure to elevated temperatures showed that NC-incorporated concrete exhibits higher compressive strength than plain concrete and that the best performance is exhibited by concrete containing 0.3 wt. % of NC. The tendency of the residual split-tensile strength of the concrete was similar to that of compressive strength. Incorporation of NC successfully maintained the loss of split-tensile strength after thermal exposure. Recently, Lee et al. [65] introduced the incorporation of NC as an admixture, to produce high-strength nano-polymer modified fireproof cementitious composites The authors produced fire resistant polymer-cement mortars (based on cement and ethylene-vinyl acetate polymer powder), varying the amount of chamotte, silica fume, and nanoclay. NC was incorporated into the initial mixes in the amounts of 1, 2, and 3 wt. %. The study showed that amounts of NC > 1 wt. % negatively affected compressive strength of specimens in the unheated state. To determine the thermal resistance of the cement mortars, their samples were exposed to 800 °C. Afterwards, statistical analysis was conducted and the tests were repeated in order to optimize the NC content in the polymer-cement mortar. The study showed that for cost and performance reasons, the amount of NC required to improve the thermal resistance of cement mortar is in the range of 0.5–1 wt. %.

**Figure 13:**
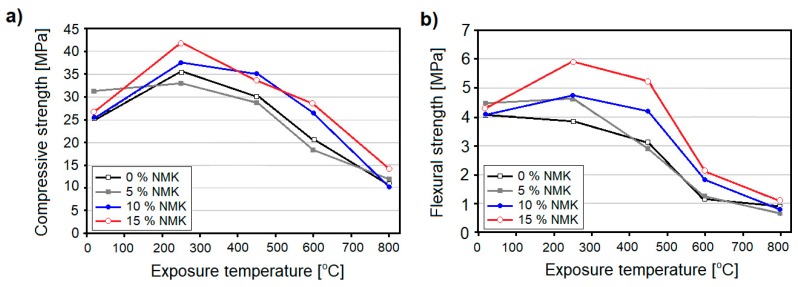
Compressive strength (**a**) and flexural strength (**b**) of cement mortars containing various amount of nano metakaolin (NMK), after thermal exposure. Reproduced with permission from [68]. Copyright Elsevier, 2012.

Morsy et al. [68] incorporated calcinated nanoclay, namely nano metakaolin (NMK), obtained from nanokaolin calcinated at a temperature of 750 °C. They analyzed the effect of NMK cement replacement in the amounts of 5, 10, and 15 wt. % of cement mortar. The pastes were exposed to temperatures of up to 800 °C. The study showed that after exposure to 250 °C, the compressive strength of all the tested samples increased, but that the highest strength increment was observed for samples containing 10 and 15 wt. % of NMK (Figure 13a). According to the authors, this effect can be attributed to the internal autoclaving effect and the pozzolanic reaction of amorphous aluminosilicate (present in NMK) with CH, during cement hydration. It is resulting in the production of additional C–S–H, with a low C/S ratio, with a calcium aluminate hydrate (C-A-H) phase being deposited in the pore system. After further thermal exposure, the samples gradually lost their strength. However, the ones containing a higher NMK content (10 wt. % and 15 wt. %) exhibited higher strength values than plain and 5 wt. % samples, up to a temperature of 800 °C. A beneficial effect of the NMK was also reflected in the flexural strength of cement mortar (Figure 13b). After exposure to 250 °C, samples containing 5, 10, and 15 wt. % of NMK exhibited strength increments by 3.4, 13.9, and 27% respectively, while plain control samples exhibited a slight strength loss. This was attributed to an increase in the amount of hydration products in the cement paste (due to an autoclaving effect), as well as because of the filler effect of NMK, which acted as a fiber in the cement matrix. Exposure to higher temperatures caused a gradual loss of flexural strength in the samples, although samples with a higher NC content (10 and 15 wt. %) underwent only a minimal decrease, up to a temperature of 450 °C. Exposure to temperatures of 600 and 800 °C caused a sharp strength loss in specimens, with samples containing NMK in the amounts of 10 and 15 wt. % exhibiting the highest resistance to elevated temperature. XRD and differential thermal analysis (DTA) confirmed the higher thermal stability of cement paste containing 15 wt. % of NMK, while SEM analysis proved that the cement matrix was less damaged (with narrower micro-cracks) after exposure to 800 °C, than the control mortar.

## 5. The Effect of Nanoalumina on the Thermal Resistance of Cement-Based Composites

Nanoalumina (NA), along with nanosilica, has gathered noticeable attention in the field of modification of cement-based composites. Due to its pozzolanic activity and high surface area, alumina particles can react with CH, forming additional hydration products such as C–A–S–H and C–A–H gel. This phenomenon is reflected in a noticeable improvement in microstructure and the mechanical characteristics of cementitious composites [41]. Accordingly, NA has been introduced as an admixture for improving the thermal resistance of cementitious composites; the studies related to this topic have been summarized in Table 3.

Heikal et al. [69] have analyzed the fire resistance of plain and superplasticized cement pastes containing NA cement replacements in the amounts of 1 wt. % and 2 wt. %, and determined the compressive strength, bulk density, total porosity and mass loss of samples exposed to temperatures up to 1000 °C. The study showed that cement paste prepared with superplasticizer exhibited better dispersion of NA and cement grains, thus resulting in a more efficient hydration process and a more homogenous cement paste microstructure. The result was cement pastes with high bulk density values as well as decreased porosities in all heating temperatures. Another positive effect of NA on the hydration process was an increase in the compressive strength of cement pastes. Specimens containing 1 wt. % NA showed the best performance among all the samples tested, under all heating temperature. This result was attributed to the enhancement of the hydration of unhydrated cement clinker (self-autoclaving effect) and an improvement in the pozzolanic reaction of NA with CH, resulting in an additional strength giving C–A–S–H phase.

**Figure 14:**
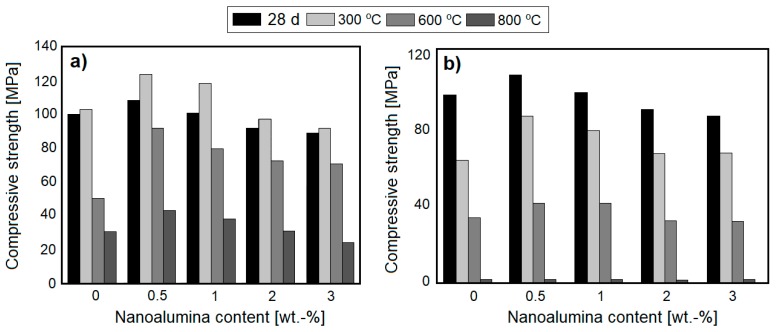
Compressive strength values of cement pastes containing nanoalumina (NA), after heating and cooling: gradually in air (**a**) and water quenching (**b**). Reproduced with permission from [41]. Copyright Springer Nature, 2017.

El-Gamal et al. [41] have evaluated the effects of 0.5, 1, 2 and 3 wt. %, NA admixture on the properties of OPC cement pastes, exposed to temperatures of 300, 600 and 800 °C. Two different cooling regimes, gradually in air and water quenching, were evaluated (Figure 14). In the case of the gradual air cooling regime (Figure 14a), all the samples tested exhibited a slight compressive strength increment, after exposure to 300 °C. Samples containing 0.5 and 1 wt. % of NA exhibited the highest strength improvement, by 15 and 18% respectively, from among all the samples tested. This effect was attributed to a self-autoclaving effect, resulting in the production of an additional amount of C–S–H phase, as well as a pozzolanic reaction leading to the production of an additional amount of C–A–H and C–A–S–H. After exposure to 600 °C, all the specimens tested exhibited a strength loss, although, samples containing NA lost only 15–21% of their initial strength, while the control samples lost around 50% of their initial strength. After exposure to 800 °C, all the samples tested exhibited a dramatic loss (more than 60%) in their initial strength. However, the mixes containing 0.5 and 1% of NA exhibited the lowest drops in strength; by 60 and 62%, respectively. In the case of the rapid cooling regime by water quenching (Figure 14b), all the testing temperatures resulted in strength losses below the initial (unheated) strength, while at a temperature of 800 °C, samples degraded after water quenching and lost their strength completely. Nevertheless, samples containing NS retained higher compressive strengths at 300 and 600 °C. The authors concluded that the optimal value for improving the fire resistance of cement pastes, is that of an NS addition of 0.5 wt. %. 

**Figure 15:**
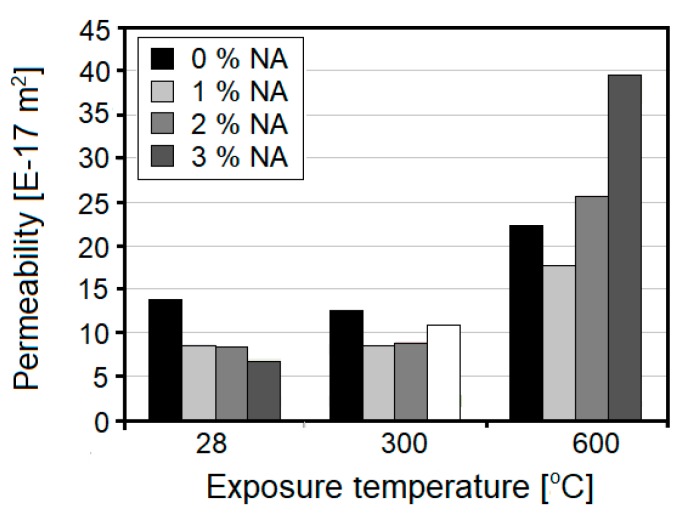
Permeability coefficients of cement mortars containing NA and control samples at 28, 300 and 600 °C. Reproduced with permission from [70]. Copyright Elsevier, 2013.

Farzadnia et al. [70] have evaluated the effects of elevated temperatures (up to 1000 °C) on cement mortars (w/c = 0.5), containing 1, 2 and 3 wt. % cement replacement. They focused on mass loss, permeability, compressive strength, elastic modulus, energy absorption, and brittleness. The authors reported that an optimal amount of NA (1 wt. %) was beneficial in improving the residual compressive strength of mortars, up to a temperature of 800 °C. The addition of 1 wt. % NA decreased the permeability (Figure 15) of samples in the unheated state, as well as after exposure to 300 °C and 600 °C, as compared to plain cement mortar. Moreover, mortars containing the optimum amount of NA (1 wt. %), exhibited a better effect on the relative modulus of elasticity, after exposure to elevated temperature, as compared to other NA modified specimens, as well as plain cement mortar. As reported by the authors, in comparison to control samples, incorporation of NA reduced the amount of CH, contributing to an improvement in the transition zone and thus a denser and stronger ITZ. Compared to C–S–H, the strength-contribution potential of CH is limited, because of its considerably lower surface area, weak Van der Waals forces and its tendency to form an oriented structure. The presence of a high amount of CH is associated with high porosity and bad durability, as it contains an extensive network of capillary pores. Thus, because it has several detrimental effects on cement mortar regarding mechanical properties and durability, reducing CH content by the incorporation of NA is considered to be advantageous. SEM analysis confirms that, in samples containing NA (1 and 2 wt. %), exposure to 400 °C resulted in fewer hairline cracks, as compared to plain cement mortar. Moreover, C–S–H in the matrix was found to have maintained its phase boundary and a less coarsened pore structure was observed.

## 6. The Effect of Nano-Iron Oxides on the Thermal Resistance of Cement-Based Composites

It has already been demonstrated that Fe_2_O_3_ as well as Fe_3_O_4_ nanoparticles positively affect the mechanical and microstructural properties of cementitious composites when incorporated [77,78,79,80]. Fe_2_O_3_ and Fe_3_O_4_ nanoparticles do not exhibit any noticeable chemical activity, and their positive effect on mechanical properties and durability is mostly attributable to their nucleation effect, as well as to the refinement of the microstructure by the nano-filling effect. The effect of nano-iron oxides on the thermal properties of cement-based composites, has been summarized in Table 3.

**Figure 16:**
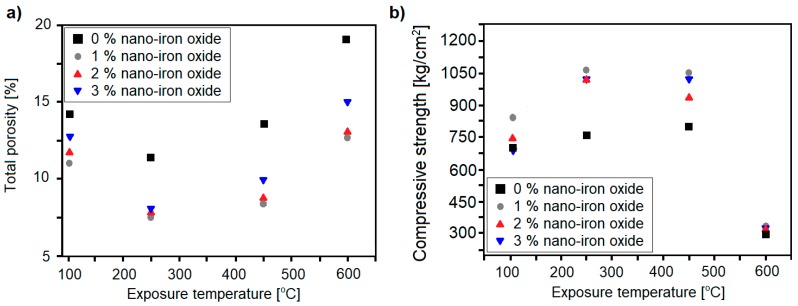
Total porosity (**a**) and compressive strength (**b**) of cement pastes containing nano-iron oxide, as a function of temperature. Redrawn from [72].

Heikal [71] analyzed the effect of Fe_2_O_3_ nanoparticles, in the amounts of 1 and 2 wt. %, on the thermal properties of cement pastes exposed to temperatures of up to 1000 °C. It was demonstrated that the incorporation of a small amount of nanomaterial (1 wt. %) is beneficial for improving the fire resistance of cement pastes modified with these nanoparticles. The incorporation of Fe_2_O_3_ nanoparticles decreased specimens’ loss in compressive strength and mass after heating. Moreover, the study showed that the presence of nano-Fe_2_O_3_ is beneficial in diminishing crack length in cement pastes. Amer et al. [72] have studied the effects of temperatures up to 800 °C, on the performance of cement pastes with 1, 2, and 3 wt. % of nano-iron oxide. Bulk density, total porosity, and the compressive strength of cement pastes exposed to elevated temperatures were studied. The study showed that the bulk density of nano-iron oxide incorporated cement pastes was higher than plain cement pastes, at all firing temperatures. As reported by the authors, this effect was attributable to the fact that iron oxide nanoparticles can act as foreign nucleation sites, accelerating C–S–H gel formation and thus leading to a more compact and dense structure. Moreover, iron oxide nanoparticles were found to exhibit a nano-filling effect, leading to a decrease in the total porosity of pastes, before and after exposure to elevated temperatures (Figure 16a). The study showed that an admixture of nano-iron oxide has a noticeable effect on strength values, after all heating temperatures, with the optimal amount being found to be 1 wt. % (Figure 16b).

## 7. The Effect of Nanotitania on the Thermal Resistance of Cement-Based Composites

Titanium dioxide (NT) nanoparticles find their application mainly in the production of photocatalytically active cement-based surfaces, due to their ability to decontaminate pollutants via photocatalytic processes [81,82]. Their effect on the hydration, mechanical properties, and durability of cement-based composites has also been extensively studied [24,83,84,85,86]. However, the incorporation of NT as an admixture for improving the thermal resistance of cement-based composites has not yet gathered significant attention (Table 3). However, an extensive study undertaken by Farzadnia et al. [73], has demonstrated its potential application for the above-mentioned purposes. They investigated the effect of NT as a cement replacement (in the amounts of 1, 2, 3 wt. %), on the compressive strength, modulus of elasticity, energy absorption, and brittleness of cement mortars (w/c = 0.35), exposed to elevated temperatures of up to 1000 °C. It was found that an optimal amount of NT (i.e., 2 wt. %) was beneficial in improving the residual compressive strength of mortars, up to a temperature of 600 °C. Nevertheless, mortars containing 1 wt. % exhibited a better effect on the relative modulus of elasticity, after exposure to elevated temperatures, than other NT-modified or plain cement mortars. Moreover, a slight decrease in the permeability of mortars modified with 1 wt. % and 2 wt. % of NT (exposed to temperatures of up to 300 °C), was also reported. At the same time, after exposure to 600 °C, all the samples containing NT exhibited increased permeability. SEM analysis showed that after exposure to a temperature of 400 °C, a less coarse pore structure was present in samples containing 1 and 2 wt. % of NT, as compared to plain cement mortar. However, after exposure to 800 °C, samples containing NT exhibited a less dense and compacted microstructure than plain cement mortar.

## 8. Discussion and Research Needs

This paper has presented a critical review of the existing research on the use of nanomaterials as admixtures for improving the thermal resistance of cementitious composites. In summary, it is clear that the use nanomaterials has an outstanding effect on the improvement of the resistance of cementitious composites to elevated temperature. The nano-sized features of nanoparticles contribute to modifications in the hydration of cement, the compaction degree of its structure, and improvement in the mechanical and fracture properties of cementitious composites, thus improving their mechanical performance and durability under elevated temperature conditions.

From all the nanomaterials examined, nanosilica has been the most extensively studied material and seems to be the most advantageous in the production of heat-resistant cementitious composites. Researchers have emphasized its beneficial influence on the hydration seeding effect, the nano-filling effect, as well as its positive role in encouraging high pozzolanic activity, resulting in the retention of strength as well as a reduction in micro-cracking. However, carbon nanotubes also have impressive mechanical properties and due to their tubular structure, exhibit bridging ability, thus restraining the possibility of cracking in cement matrices, under thermal stress. Furthermore, other carbon-based nanomaterials have also come up in recent research works; for instance, graphene oxide, though its incorporation requires further investigation. Other nanomaterials, such as nanoclay, nanoalumina, nano-iron oxides, and nanotitania can also be used in cement-based composites. However, only a few studies have been carried out in regard to utilizing these nanomaterials for improving the thermal resistance of composites. Therefore, more research is required on the potential effects of these nanoparticles on the thermal resistance of cementitious composites.

The following research needs can be drawn from the review presented above:

1. There is a lack of comprehensive, comparable work analyzing the properties of nanomaterials, with similar mix designs, heating and cooling regimes and testing procedures, which would make it possible to establish which nanomaterials have the most beneficial performance, in terms of improving the thermal resistance of cementitious composites. In addition, studies regarding some nanomaterials are very limited, and hence some findings from different studies are often mutually exclusive or not fully explored.
2. Most studies are related to performance in cement pastes or cement mortars, with only very few studies available regarding the thermal resistance of normal strength and high-strength concretes. In addition, the effects of nanomaterials on the cracking and spalling potential of cement-based composites are still undetermined. It is crucial that this issue addressed while considering the further incorporation of nanomaterials in cementitious composites with compacted and dense matrixes, such as HSCs.
3. No studies regarding optimization of mix design, by combining nanomaterials with other SCMs, enabling a simultaneous decrease in production costs as well as an improvement in the performance of cementitious composites under elevated temperature conditions, are available. In addition, no evaluation of a potential combination of various nanomaterials, or incorporation of molecular hybrids (i.e., core-shell structures) has yet been presented.
4. Most of the available studies evaluate the performance of nanomaterials based on laboratory results. Therefore, it is necessary to verify performance in real scale tests, to obtain practical experience in the application of nano-materials, thus enabling their further application in industry.
5. Evaluation of the long-term post-fire performance and durability of nano-modified cement-based composites is absent. Most of the studies available are related to the effects of nanoparticles on mechanical properties (mainly compressive strength), while less work has been devoted to studying other properties, such as permeability, water absorption or porosity, after thermal exposure.

To extract the full benefits of the incorporation of nanomaterials, future research should address the abovementioned issues.

## Figures and Tables

**Table 1:** The effects of temperature on physico-chemical, visual, and mechanical properties of cement-based composites exposed to elevated temperatures. Based on: [1,2,3,5,9,10,13].

Temperature Range	Physico-Chemical Changes	Effect on Cracking	Effect on Strength
20–100 °C	Ettringite dehydration (from 80 to 150 °C Evaporation of capillary and free water	No cracking is observed	No effect or slight strength improvement
100–200 °C	Dehydration of calcium aluminosulphate hydrates (120–140 °C) Gypsum decomposition (150–170 °C) Beginning of loss of stability of cement paste Loss of C–S–H interlayer water	No cracking is observed	No effect or slight strength improvement
200–400 °C	Break of some siliceous aggregates (approx. 350 °C) Dehydration of C–S–H phase	First noticeable cracks are developed	<300 °C no effect or slight strength improvement. >300 °C strength loss by 15–40%
400–600 °C	Decomposition of hydration products Decomposition of Ca(OH)_2_ to CaO + H_2_O (450–550 °C) Destruction of C–S–H gel Quartz phase transformation (573 °C) from α-quartz to β-quartz assisted with increment of volume	Intensification of cracking in aggregate, cement paste and ITZ	Strength loss by 40–60%
600–800 °C	Loss of load-bearing capacity Second phase of C–S–H decomposition and formation of β-C_2_S at 600-800 °C Decomposition of poorly crystalized CaCO_3_ Decarbonization of limestone aggregate	Severe cracking, ITZ cracks, starting of collapsing of structural integrity of concrete	Strength loss by 80%
800–1000 °C	Breakdown of C–S–H phase Total loss of water of hydration∙ Ceramic binding	Loss of bond between aggregate and paste	Loss of compressive strength or almost all of compressive strength is lost

**Table 2:** Summary of available studies related to the effects of nanosilica (NS) on the thermal resistance of cement-based composites.

Size [nm] (Surface Area [m^2^/g])	Amount [wt. %]	Heating Temperature [°C]	Type of Material	Tested Mechanical Properties	Ref.
15	1, 4	200–400–650–850–950	Cement paste	Compressive strength	[40]
30 ± 5 (195)	0.5, 1, 2, 3	300–600–800	Cement paste	Compressive strength	[41]
30 nm (180–300)	5	100–200–300–400–500	Cement paste	Compressive strength	[42]
12–15 (n/a)	1.5, 3	500–800	Cement paste and cement mortar	Compressive strength	[43]
n/a	2.5, 5, 7.5	400–700	Cement mortar	Flexural strength, compressive strength	[44]
n/a	2.5, 5, 7.5	400–700	Cement mortar	Flexural strength, compressive strength	[45]
n/a	1, 2, 3, 4, 5	200–400–600–800	Cement mortar	Flexural strength, compressive strength	[28]
30-70 (n/a)	3	200–400–600–800	Concrete	Compressive strength, split-tensile strength	[39]
45 (60)	1.5, 3, 4.5	400–600–800	Concrete	Compressive strength, tensile strength	[46]
16–20 (170–200)	5	200–500–800	Concrete	Compressive strength	[47]
5–8 (n/a)	1, 2, 3, 4, 5	100–200–400–600–800	Concrete	Compressive strength	[48]
n/a	1, 2, 3, 4, 5	200–400–600	Concrete	Flexural strength, compressive strength	[49]
30 (200 ± 10)	2	200–400–600–800	Concrete	Uniaxial tensile strength	[50]
30 (200 ± 10)	2	200–400–600–800	Concrete	Compressive strength, tensile splitting strength, flexural strength	[51]

**Table 3:** Summary of available studies related to the effect of various nanomaterials on thermal resistance of cement-based composites.

Type of Nanomaterial	Size and Specific Surface Area (SSA)	Amount [wt. %]	Heating Temperature [°C]	Type of Material	Tested Mechanical Properties *	Optimal Amount [wt. %]	Ref.
MWCNT	10–40 nm (diameter), 5–10 μm (length), 93.81 m^2^/g (SSA)	0.02, 0.05, 0.1, 0.2	300 - 600–800	Cement paste	F_c_	0.1	[59]
Carbon nanosphere-carbon fiber	85 nm CNS on 5–6 mm CF	0.55	200–400–600	Cement paste	F_c_	-	[61]
Hydroxylated MWCNT	>50 nm (diameter), length 20 µm, <400 (aspect ratio), >40 m^2^/g (SSA)	0.1, 0.2	200–400–600	Cement paste	F_f_, F_c_	0.2	[63]
Graphene oxide	n/a	n/a	400–600–800	Concrete	F_t_, F_c_	-	[62]
Graphene sulfonate nanosheet	1–2 nm (diameter), 50–100 µm (length)	0.1	200–400–600–800–1000	Concrete	F_c_, F_st_	-	[60]
Nanoclay	1 nm (diameter),300–500 (aspect ratio)	1, 2	200–400–600	Cement mortar	F_f_, F_t_, F_c_	2	[64]
Nanoclay	n/a	1, 2, 3	800	Cement mortar	F_c_	0.5-1	[65]
Nanoclay	10 nm (diameter), >600 m^2^/g (SSA)	0.1, 0.3, 0.5	400–440–580–800	Concrete	F_c_, F_st_	0.3	[66]
Nanoclay	<10 nm (diameter)	0.1, 0.3, 0.5	300–440–500–580–800–1000	Concrete	F_c_	0.5	[67]
Nanometakaolin	100·50·10 nm, 48 m^2^/g (SSA)	5, 10, 15	250–450–600–800	Cement paste	F_c_	15	[68]
Nanoalumina	40 ± 5 nm, 173 m^2^/g (SSA)	0.5, 1, 2, 3	300–600–800	Cement paste	F_c_	0.5	[41]
Nanoalumina	15 ± 3 nm, 165 ± 12 m^2^/g (SSA)	1, 2	250–450–600–800–1000	Cement mortar	F_c_	1	[69]
Nanoalumina	13 nm, 85–115 m^2^/g (SSA)	1, 2, 3	100–200–300–400–600–800–1000	Cement paste	F_c_	1	[70]
Nano-iron oxide	10–20 nm, 50.5 m^2^/g (SSA)	1, 2	200–300–400–600–800–1000	Cement paste	F_c_	1	[71]
Nano-iron oxide	14.6 nm	1, 2, 3	105–250–450–600–800	Cement paste	F_c_	1	[72]
Nanotitania	21 nm, 35–65 m^2^/g (SSA)	1, 2, 3	100–200–300–400–600–800–1000	Cement mortar	F_c_	2	[73]

* F_c_—compressive strength, F_f_—flexural strength, F_t_—tensile strength, F_st_—split-tensile strength.

## Abbreviations

C–S–H	calcium-silicate-hydrates
CH	calcium hydroxide (portlandite)
β-C_2_S	β-dicalcium silicate
ITZ	interfacial transition zone
OPC	ordinary Portland cement
HSC	high strength concrete
SFRC	steel-fibre reinforced concrete
SCM	supplementary cementitious material
SF	silica fume
GGBS	ground granulated blast-furnace slag
FA	fly ash
NS	nanosilica
NA	nanoalumina
NC	nanoclay
NMK	nanometakaolin
NT	nanotitania
CNT	carbon nanotube
MWCNT	multi-walled carbon nanotube
CF	carbon fiber
CNF	carbon nanofiber
GO	graphene oxide
GSNS	graphene sulphonate nanosheet
MIP	mercury intrusion porosimetry
XRD	X-ray diffraction
DSC	differential scanning calorimetry
SEM	scanning electron microscopy
